# A Study about Schottky Barrier Height and Ideality Factor in Thin Film Transistors with Metal/Zinc Oxide Nanoparticles Structures Aiming Flexible Electronics Application

**DOI:** 10.3390/nano11051188

**Published:** 2021-04-30

**Authors:** Ivan Rodrigo Kaufmann, Onur Zerey, Thorsten Meyers, Julia Reker, Fábio Vidor, Ulrich Hilleringmann

**Affiliations:** 1 Sensor Technology Department, University of Paderborn, 33098 Paderborn, Germany; ozerey@t-online.de (O.Z.); meyers@sensorik.upb.de (T.M.); reker@sensorik.upb.de (J.R.); ulrich.hilleringmann@uni-paderborn.de (U.H.); 2 CAPES, Coordenação de Aperfeiçoamento de Pessoal de Nível Superior, Brasilia 70040-020, Brazil; 3 Departamento Interdisciplinar, Universidade Federal do Rio Grande do Sul (UFRGS), Tramandaí 95590-000, Brazil; fabio.vidor@ufrgs.br

**Keywords:** thin film transistors, flexible electronics, zinc oxide nanoparticles, metal-semiconductor-metal, Schottky contact, Schottky barrier height

## Abstract

Zinc oxide nanoparticles (ZnO NP) used for the channel region in inverted coplanar setup in Thin Film Transistors (TFT) were the focus of this study. The regions between the source electrode and the ZnO NP and the drain electrode were under investigation as they produce a Schottky barrier in metal-semiconductor interfaces. A more general Thermionic emission theory must be evaluated: one that considers both metal/semiconductor interfaces (MSM structures). Aluminum, gold, and nickel were used as metallization layers for source and drain electrodes. An organic-inorganic nanocomposite was used as a gate dielectric. The TFTs transfer and output characteristics curves were extracted, and a numerical computational program was used for fitting the data; hence information about Schottky Barrier Height (SBH) and ideality factors for each TFT could be estimated. The nickel metallization appears with the lowest SBH among the metals investigated. For this metal and for higher drain-to-source voltages, the SBH tended to converge to some value around 0.3 eV. The developed fitting method showed good fitting accuracy even when the metallization produced different SBH in each metal-semiconductor interface, as was the case for gold metallization. The Schottky effect is also present and was studied when the drain-to-source voltages and/or the gate voltage were increased.

## 1. Introduction

Integrated electronic devices produced on flexible and transparent substrates are responsible for a number of innovative modern products, such as displays, radio frequency identification (RFID) tags, wearable electronics, and sensors. Moreover, the development of a new chain employing these devices is related to the emergence of the concept of the Internet of Things (IoT), where it will be possible to connect multiple objects across the world wide web [1]. Faced with this reality, flexible electronics have received significant attention over the last decade from both research and industry groups around the world. The main reasons are the possibility of innovation, low-cost manufacturing, large-area processing solutions, and compatibility with large-scale printing [2,3,4,5].

Thin Film Transistors (TFTs) are active devices switching driving electrical currents in flexible microelectronic systems. As a semiconductor material for TFTs, zinc oxide (ZnO) has attracted attention by showing outstanding electrical, chemical, and sensory characteristics [6,7]. ZnO has a direct bandgap of about 3.3 eV at room temperature, turning it into a transparent material to the visible light spectrum. Furthermore, it is possible to produce ZnO at low temperatures (~150 °C) [8], lowering the thermal budget time to process TFTs.

Despite some promising results concerning cost-efficient flexible electronics with ZnO nanoparticles (NP) [9], some challenges regarding the contact resistance between the source/drain electrodes and the semiconductor have to be overcome. The contact between metal and semiconductor generates a barrier in energy, the so-called Schottky barrier. A high contact resistance results in a contact-limited charge carrier injection, thus limiting the device performance drastically. Klauk [10] presents an important approach regarding the efforts on reducing this Schottky barrier, decreasing the channel size, and minimizing parasitic capacities of source and drain coupling with the gate electrode. From the study and development of techniques and materials to overcome such challenges, flexible in/organic electronics can thus be able to reach applications across the MHz barrier for the next generation of TFTs.

Although many studies focus on common transistor parameters such as charge mobility (µ_e_ for electrons), subthreshold swing (SS), turn-on voltage (V_on_), threshold voltage (V_th_), and the ratio of the current in the on and off state (I_on_/I_off_) [4], less deal with the Schottky contact and how it affects TFT performance.

In this article, we present a study of how the Schottky contact between different metals (aluminum, gold, and nickel) and the ZnO NP n-type layer affects the TFT operation. A Metal-Semiconductor-Metal (MSM) model was considered, and with simulation tools, we were able to fit and extract information about the Schottky Barrier Height (SBH) and about ideality factors (n) as a function of the gate voltage and/or electric field generated by the drain-to-source voltage.

## 2. Materials and Methods

**Figure 1:**
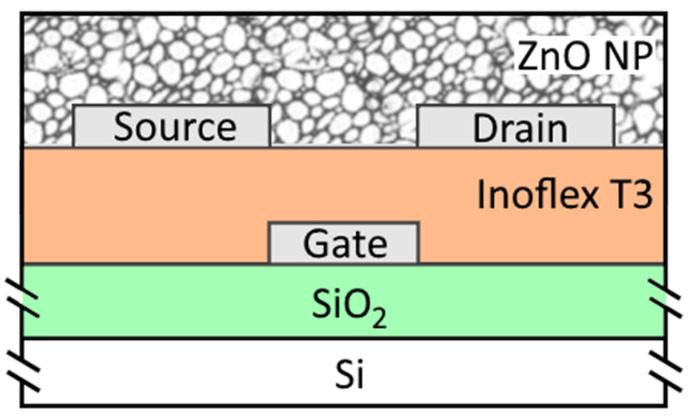
Cross-section view of the fabricated TFTs. Al, Au, and Ni were used for source and drain contacts.

The TFTs were prepared over a SiO_2_ (700 nm)/Si wafer with an inverted coplanar setup (Figure 1). The wafer itself served as a rigid substrate for the present study purpose, although the TFT structures could be integrated on a transparent and flexible substrate in order to evaluate other parameters, like light transmittance and flexibility. In this manner, all the experiments were performed at the maximum temperature of 150 °C, simulating the thermal treatments for polymeric substrates. Over the SiO_2_ layer, 50 nm of aluminum followed by 7 nm of titanium were evaporated under vacuum conditions. A gate pattern definition was performed using standard contact photolithography and wet etching processes. An organic-inorganic nanocomposite was used as a material for the gate dielectric (available from Inomat GmbH, trade name: Inoflex T3 [11]), based on hydrolyzed and condensed acrylate functionalized silane. TiO_2_ nanoparticles were added by co-condensation to increase the permittivity of the Inoflex T3 (ε_r_ = 10 at 50 Hz). The final thickness of the spin-coated gate dielectric was in the range of 150–180 nm. Thermal processes were also performed for cross-linking and curing in at 115 °C air temperature for 30 min followed by a UV exposure for a total of 4 min.

For the source and drain contacts, aluminum (Al), gold (Au), and nickel (Ni) were used (work function around 4.2 eV, 4.8 eV, and 4.9 eV, respectively) [12]. These metals were evaporated and patterned following standard contact photolithography and lift-off processes, with thicknesses of 150 nm for Al and Ni, and 100 nm for Au. An aqueous ZnO NP dispersion was deposited over the TFTs template using the doctor blade technique [13], forming a homogenous film covering the source and drain contacts and so filling the channel region. Moreover, a thermal process at an air temperature of 115 °C for 30 min was performed for water evaporation. A UV exposure of 4 min in total and a humidity treatment were also conducted in order to release O_2_ molecules and adsorb H_2_O molecules on the ZnO NP surface, increasing the film conductivity [14]. For each set of metal, the channel length (source to drain distance) of 3 µm and 5 µm were considered, named L3 and L5, respectively. The width for both channel lengths was W = 1000 µm.

The TFTs were electrically characterized at room temperature in a dark and electrically isolated environment using a parameter analyzer Agilent 4156A.

## 3. TFT Electrical Parameters

**Figure 2:**
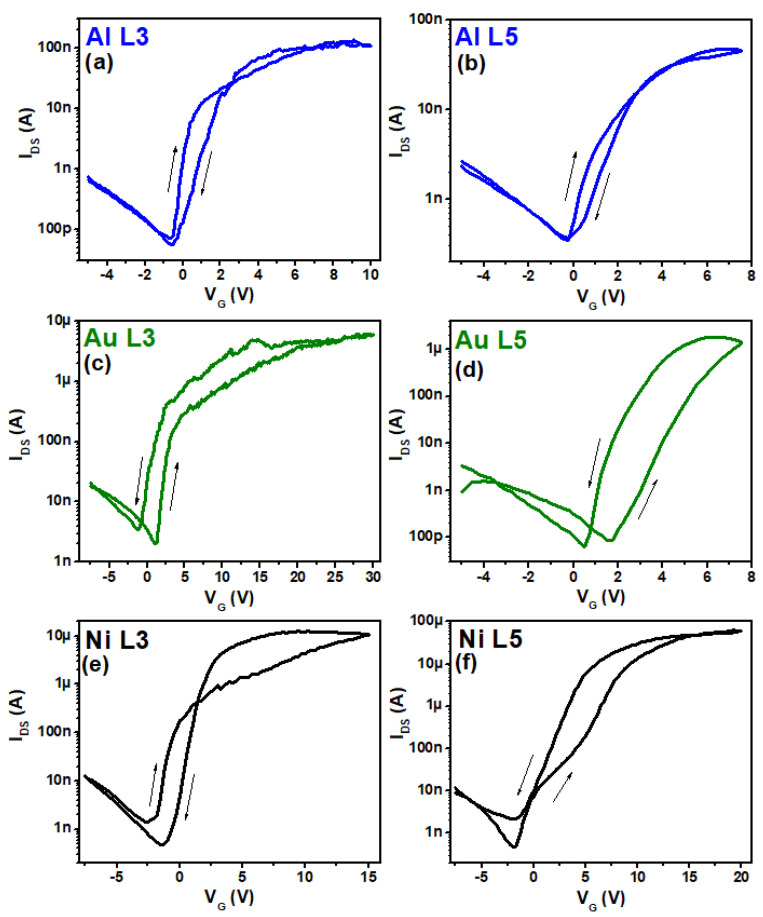
Forward and backward TFT transfer curves with V_DS_ = 5 V applied for: (**a**) Al for the drain and source metallization with channel length L = 3 μm; (**b**) Al with L = 5 μm; (**c**) Au with L = 3 μm; (**d**) Au with L = 5 μm; (**e**) Ni with L = 3 μm; (**f**) Ni with L = 5 μm.

The TFT performance usually can be evaluated in terms of some common electric parameters as µ_e_, SS, V_on_, V_th_, and I_on_/I_off_. These parameters can be affected by the choice of gate dielectric and semiconductor materials and metals used for the gate and source/drain contacts. The metals chosen for source and drain terminals are especially highlighted in this study. These metals in contact with the ZnO NPs create an SBH that directly affects the TFT performance. Figure 2 shows the transfer characteristic curves (I_DS_ vs. V_G_) applying a source to drain voltage V_DS_ of 5 V for all the TFTs that were measured in this work. A summary of the results extracted from Figure 2 are presented in Table 1. For the extraction of the mobility, the transfer characteristics were necessary, from which the transconductance was obtained. Together with other parameters such as the dielectric capacitance, the length and width of the transistor’s channel, and the drain voltage, the value for the mobility could be evaluated. The Ni TFTs presented the best results. Ni L5 showed an I_on_/I_off_ of 1.3 × 10^5^ and mobility of 0.151 cm^2^ V^−1^ s^−1^, despite the fact that it presented a higher SS and a more negative V_on_ voltage in comparison to the other TFTs. The Au TFTs showed intermediate results, being the only one with a positive V_on_. The Al TFTs in this configuration did not have the expected characteristics, especially for I_on_/I_off_ and mobility (the last one is in the range of 10^−4^ cm^2^ V^−1^ s^−1^). Specifically to the Al samples, it is well-known that the employment of inverted coplanar setup jeopardizes the contact area between the drain and source electrodes and the active semiconducting layer [15,16]. Additionally, the lift-off techniques used in the integration process could induce chemical stress at the dielectric-semiconductor interface [17].

**Figure 3:**
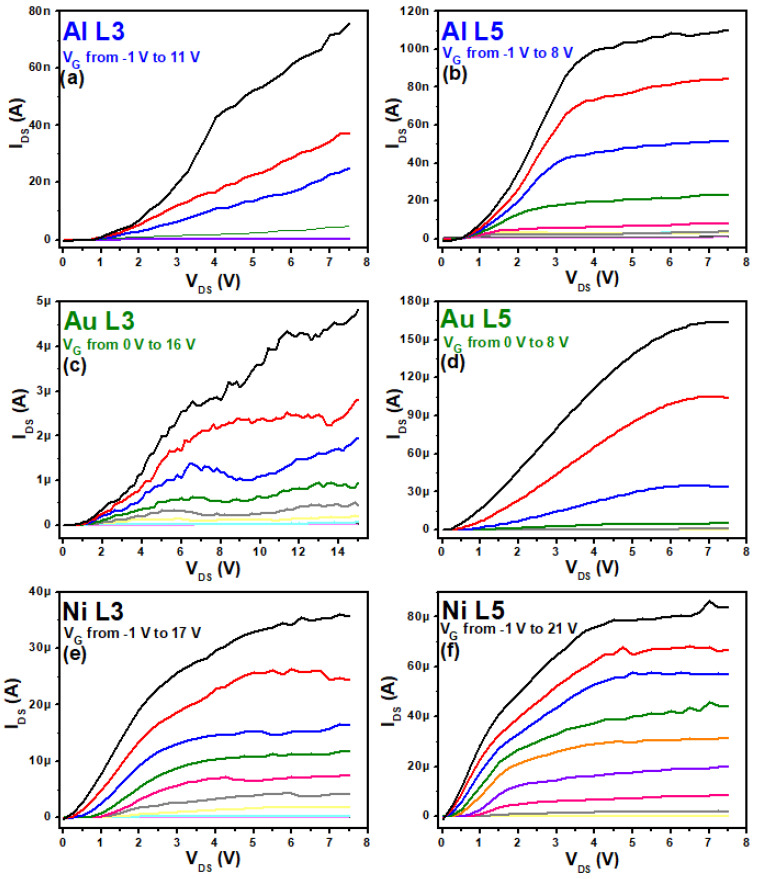
Corresponding output curves of the TFTs from Figure 2 with different gate voltage (V_G_) applied for: (**a**) Al for the drain and source metallization with channel length L = 3 μm; (**b**) Al with L = 5 μm; (**c**) Au with L = 3 μm; (**d**) Au with L = 5 μm; (**e**) Ni with L = 3 μm; (**f**) Ni with L = 5 μm.

The output characteristic curves are shown in Figure 3. The first noticeable aspect of these curves was that Ni TFTs presented a saturation current for higher V_DS_ in all the ranges of the gate voltage (V_G_) applied, from depletion to strong accumulation (negative to positive V_G_). In addition, the Ni TFTs depicted the highest channel current on accumulation mode. The Au transistors presented with an intermediate behavior, yet with no clear saturation current established. For a fixed V_G_ value of 8 V and in the saturation region of the L5 TFTs, the highest I_DS_ was for Ni (~5 µA), followed by Au (~0.6 µA) and Al (~0.1 µA). An s-shape was also observable for the Al and Au TFTs, indicating a high contact resistance between metal and semiconductor [18].

From the previous analysis, TFTs with Ni source and drain metallization showed the best results, followed by Au and Al, respectively. The current injected from source/drain into the n-type channel plays an important role in better understanding TFTs behavior. For this reason, a more detailed study regarding the current in Metal-Semiconductor-Metal (MSM) structures is required [10].

## 4. Metal-Semiconductor-Metal (MSM) Measurements

**Figure 4:**
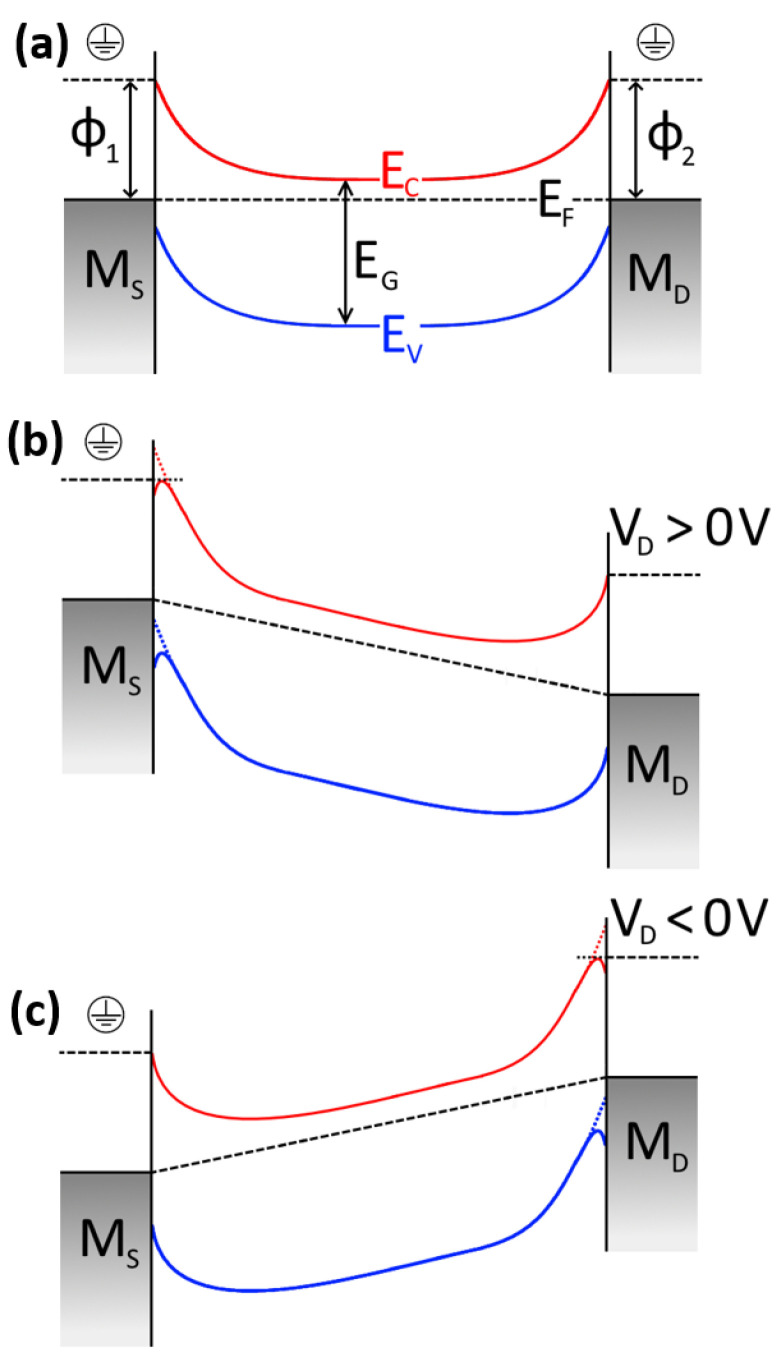
Metal-Semiconductor-Metal band diagram for: (**a**) both Schottky junctions at ground potential, (**b**) M_S_ (source metal) in ground potential and M_D_ (drain metal) with a positive voltage applied, and (**c**) M_S_ in ground potential and M_D_ with a negative voltage applied. E_C_, E_V_, E_F_, and E_G_ represent the conduction band, valence band, Fermi level band, and the semiconductor bandgap, respectively.

The electrical current in a TFT structure can be understood as a flow of negative charges going from one metal (drain) electrode into the channel and then being collected by the other metal (source) electrode. This means that in the TFTs, a Metal-Semiconductor-Metal (MSM) structure exists with one Schottky diode at each interface. Figure 4a shows the band diagram with no voltage applied in the source (M_S_) and drain (M_D_) metals. When metal and semiconductors were in contact, the semiconductor Fermi level aligned with the metalwork function. A Schottky barrier appeared in each interface, and electrons faced this energy barrier when one of the diodes was reverse biased. In fact, what limits the current in TFTs is the diode that is reverse biased [19]. Figure 4b,c illustrates the case when M_D_ has positive and negative voltages applied, respectively (note that M_S_ is ground potential). In Figure 4b, the diode in the M_S_ interface was reverse biased, and the diode in the M_D_ interface was direct biased, and the contrary is presented in Figure 4c. The equation that regulates the current I(V_DS_) in TFTs can thus be written as in [19,20]:

(1) $$I\left(V_{\mathrm{DS}}\right)=\frac{I_{1}I_{2}\sinh\left(\frac{{\mathrm{qV}}_{\mathrm{DS}}}{2k_{B}T}\right)}{I_{1}\exp\left(-\frac{{\mathrm{qV}}_{\mathrm{DS}}}{2n_{1}k_{B}T}\right)+I_{2}\exp\left(\frac{{\mathrm{qV}}_{\mathrm{DS}}}{2n_{2}k_{B}T}\right)}$$

where the saturation currents I_1_ and I_2_ are given by:

(2) $$I_{1}\left(T\right)=S_{1}A^{*}T^{2}\;\exp\left(-\frac{\Phi_{1}}{2k_{B}T}\right)$$

(3) $$I_{2}\left(T\right)=S_{2}A^{*}T^{2}\;\exp\left(-\frac{\Phi_{2}}{2k_{B}T}\right)$$

In the above-mentioned equations, k_B_ is the Boltzmann constant, T is the absolute temperature in Kelvin, S_1_ and S_2_ are the diodes areas (thickness vs. width) in cm^2^, A* is the effective Richardson constant (A* = 32 A cm^−2^ K^−2^ for ZnO NP [21]), Φ_1_ and Φ_2_ are the SBH in eV, n_1_ and n_2_ are the ideality factors for each diode junction and V_DS_ is the drain-to-source voltage difference. The ideality factor gives information about the current mechanism involved in the Schottky junction. An ideality factor close to unity means that all the current is generated by thermionic emission. Values above unity reveal that other current mechanisms are involved, like tunneling, recombination, and diffusion of electrons or holes [22].

The gate voltage V_G_ can also regulate the total current in a TFT. In this case, the saturation currents I_1_ and I_2_ can be modulated depending on the V_G_ applied, and hence the total current is a function not only of V_DS_, but also of V_G_: I (V_DS_, V_G_) [23].

As the total current in TFTs is limited by the diode that is reverse biased, it is important that the SBHs are as low or thin as possible, reproducing ohmic contacts in both Schottky junctions. Indeed, when the TFT is in the on state, it is essential that it conducts the maximum current possible with the lowest V_DS_ bias applied. Still, the gate electrode takes control over the TFT between the on and off state, accumulating or depleting the channel and so modulating the operation.

**Figure 5:**
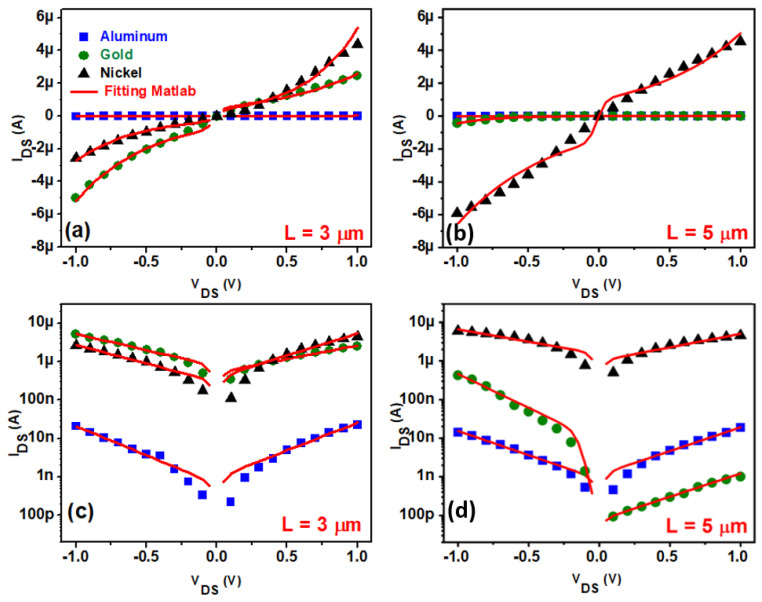
Electrical current through Metal-ZnO NP-Metal structures as a function of the drain to source voltage applied in (**a**,**b**) linear and (**c**,**d**) log scale. The gate voltage VG was fixed to 4 V for all the measurements. Al, Au, and Ni were the metals used for drain and source metallization. The red line is a Matlab simulated fitting curve considering the Equations (1)–(3). TFTs with channel lengths of L = 3 μm and 5 μm were evaluated.

For all the TFTs (Al, Au, and Ni, L = 3 and 5 µm) the output curves I_DS_ vs. V_DS_ with different V_G_ were measured, both in the positive and negative range of V_DS_. Using Equations (1)–(3) it was possible to fit the model to all experimental data and to extract information about SBH and ideality factors for both diodes of each TFT. Figure 5a,b presents a plot of the experimental points and the simulation lines of I_DS_ vs. V_DS_, with V_DS_ ranging from −1 to 1 V and V_G_ fixed at 4 V. For the fitting, the numerical computing software MATLAB (version R2018b) was used [24]. For the sake of clarity, Figure 5c,d depict the same results for Al L = 3 µm, Al L = 5 µm and Au L = 5 µm; however, in log scale for a better observation of the fitting curves. As can be seen, the fitting model was in good accuracy with the experimental points for all the source/drain metals used. A coefficient of determination R^2^ around 0.97 ± 0.03 was evaluated for all simulations in this work. Figure 5 shows that the Al TFTs presented the lowest current among the metals, which was around two orders of magnitude lower than for Ni TFTs. Ni and Au TFTs presented close current values for L = 3 µm, but the Ni transistor in L = 5 µm had a higher current for both positive and negative V_DS_ bias.

**Figure 6:**
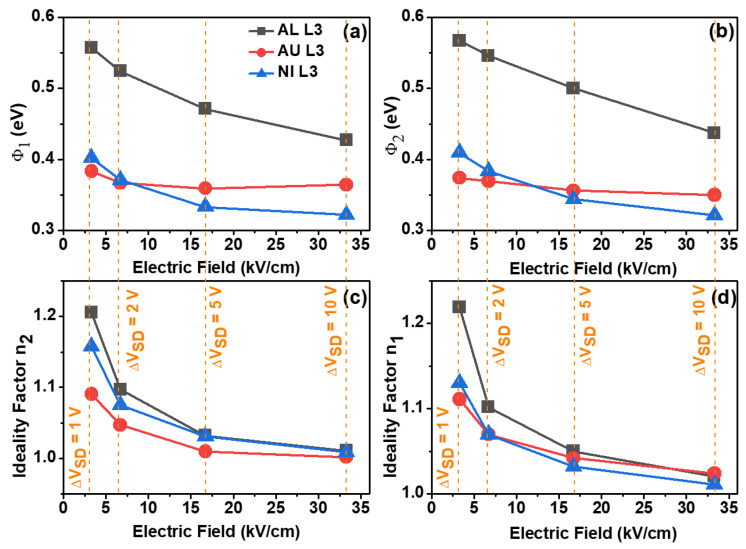
SBHs and ideality factors as a function of the electric field inside the TFTs channel with L = 3 μm, created by the potential difference between source and drain metals (∆V_DS_ = 1, 2, 5, and 10 V). (**a**,**b**) show the Schottky Barrier Height 1 and 2, (**d**,**c**) show the ideality factor n_1_ and n_2_.

**Figure 7:**
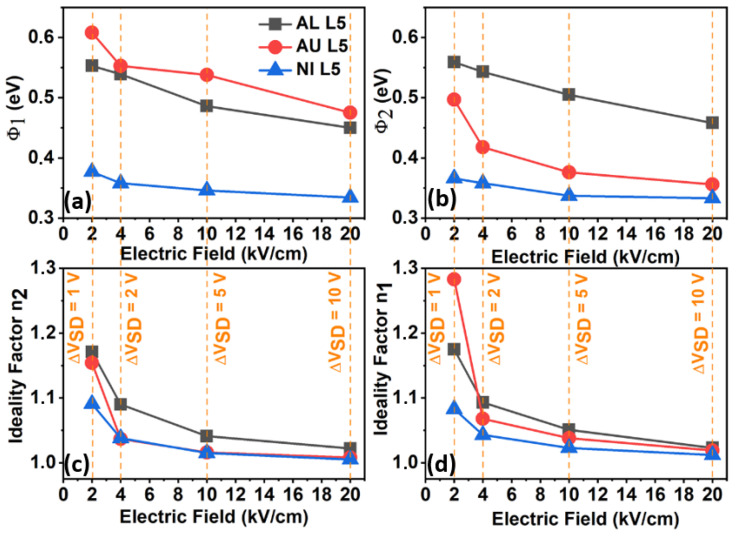
SBHs and ideality factors as a function of the electric field inside the TFTs channel with L = 5 μm, created by the potential difference between source and drain metals (∆V_DS_ = 2, 5, and 10 V). (**a**,**b**) show the Schottky Barrier Height 1 and 2, (**d**,**c**) show the ideality factor n_1_ and n_2_.

Although Figure 5 presents the result for V_DS_ ranging from −1 to +1 V, we have also measured and implemented the fitting for the data of V_DS_ going from −2 to +2 V, −5 to +5 V, and −10 to +10 V. The SBHs and ideality factors were then extracted and plotted in Figure 6 and Figure 7. The Schottky barrier heights Φ_1_ and Φ_2_ were obtained by the saturation currents from the reverse diodes related to Equations (2) and (3), respectively. For the positive and negative V_DS_ range, information about Φ_1_ and Φ_2_ were obtained, respectively. The ideality factors, on the contrary, were mainly correlating with the direct bias diodes. In this way, n_1_ was strongly correlated to the negative V_DS_ range and n_2_ with the positive range.

Figure 6 shows the results of the SBH and the ideality factors as a function of the electric field modulus created by the potential difference between source and drain (∆V_DS_ = 1, 2, 5, and 10 V) for TFTs with L = 3 µm. For these measurements, a fixed V_G_ of 4 V was applied. For both SBH, the Al transistor showed the highest values, ranging from 0.56 to 0.44 eV accordantly to the electric field increase. Au and Ni TFTs had closer SBH values, ranging from 0.40 to 0.34 eV. Yet, for the higher electric field, the Ni TFT presented with the lowest SBH of 0.32 eV.

The SBH decreasing with a higher electric field is well known as the Schottky effect (or image force lowering effect) [25]. This effect is also represented in Figure 4b,c, in the interface of the diode that was reverse biased, causing the conduction and valence bands to be curved. As the SBH at the reverse diode was decreased by the Schottky effect, it was also expected that the thermionic emission should be higher through the lowered SBH. The ideality factor should also decrease with increasing electric field. Indeed, this was what was observed in Figure 6c,d. Even for the lowest ∆V_DS_ = 1 V, both ideality factors for all source/drain metals were n = 1.15 ± 0.05. As the electric field increased, both ideality factors were close to unity. In general, the ideality factor for the Al TFT was slightly higher than for Au and Ni (up to 0.1 higher with the smaller electric field).

Figure 7 shows the results for the TFTs with L = 5 µm. The results for Al and Ni source/drain metals were in close agreement with the results presented in Figure 6. For these samples, the channel length had no influence on the Schottky diodes. For Au L5, there was a significant difference between Φ_1_ and Φ_2_ and when compared to the results presented in Figure 6b. Figure 5d shows the logarithmic scale of I_DS_ vs. V_DS_ for the Au L = 5 µm TFT. In fact, the I_DS_ was not symmetric for the positive and negative V_DS_ range as it was for the other metals. Therefore, an asymmetry of the SBH was expected, as shown in Figure 7. This asymmetry is still under investigation, and eventually, it might be found to be related to the memristor effect [26,27]. Even though the results of an inverted staggered TFTs setup are not presented in this work, this effect could also be observed in this kind of structurem, and it seems to be even more prominent.

**Figure 8:**
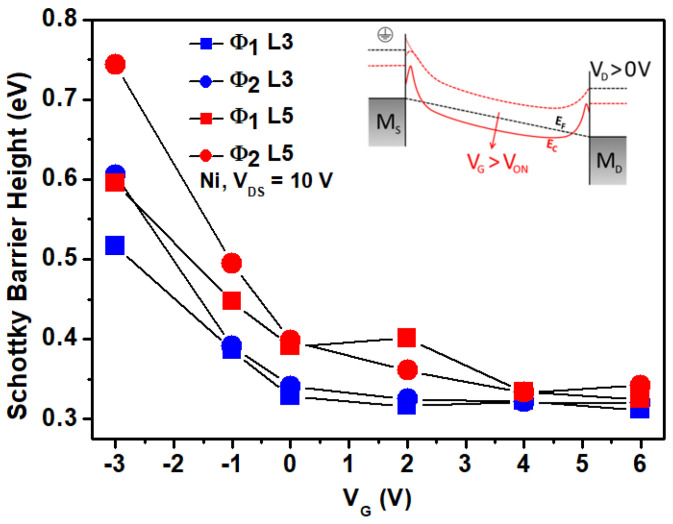
Schottky barrier height for both source and drain Ni contact with ZnO NP, as a function of the gate voltage V_G_. As V_G_ increased for positive values, the Schottky effect was more prominent and thus reduced the SBH. The inset figure represents the band diagram of an MSM structure under different gate voltages, elucidating the Schottky effect.

The Schottky effect also appears when the gate voltage V_G_ varies [23]. Figure 8 shows the SBH as a functions of V_G_ for the Ni sample with V_DS_ = 10 V. Both the Φ_1_ and Φ_2_ of L = 3 and 5 µm are shown. As V_G_ increases for positive voltage, both SBHs decreased and tended to a value of around 0.32 eV. For the negative V_G_, the channel was in depletion mode and presented a higher and non-convergent SBH value. The inset of Figure 8 helps to elucidate this phenomenon. Not only did the drain-to-source voltage cause the Schottky effect to appear in the reverse diodes, but it also occurred when the gate voltage induced more electrons into the channel (for an n-type semiconductor). This effect was more prominent in both interfaces, independent of the diode being in the reverse or in the direct bias condition. With V_G_ > V_on_, more electrons were induced into the channel, and the SBH width became thinner on both metal interfaces. Those electrons closer to the interface with the metals increasd the Schottky effect, causing the SBHs to be lowered with higher V_G_ [23,25].

With a high electric field in the channel or with a high V_G_ applied, it seems that for Ni source/drain metal; the SBH tended to a value close to ~0.3 eV. The Fermi-level pinning could fix the SBH to this value, but it is unclear if this is the case. A possible explanation might be that interface layers (like non-stoichiometry interfacial layers) cause the Fermi-level depinning [28]. The work function of the metal surface can also be different from those known by a completely clean surface. In this matter, more measurements varying the temperature should be performed.

For a more precise Schottky analysis, the mathematical equations used for the Matlab simulation tool should also consider other charge transport mechanisms, especially tunneling through a possible thin insulator layer between metal and ZnO NP [29] or traps at the metal/semiconductor interface and semiconductor bulk. As the barrier heights of metal/semiconductor systems are influenced by the metalwork function and by the interface states [25], the current mechanism through traps could play an important role in the TFT channel current. Although previous studies addressing traps in ZnO NPs TFTs were performed by the authors [14,15,30], how the interface states at the contacts and the presence of traps may influence the SBH, and thus the channel current remains an open question. Further studies regarding traps in TFTs are being carried out in order to have a better overall understanding of the charge carrier transport mechanism at these contacts, as well as in the semiconducting layer. Nevertheless, the model and the simulation tool proposed in this article describe in an accurate form the rule of the Schottky barriers and ideality factors in the charge transport mechanism that takes place in the interface of the MSM structures of the TFTs.

## 5. Conclusions

Thin-film transistors (TFTs) with zinc oxide nanoparticles (ZnO NP) as semiconductor material were fabricated and characterized. Aluminum, gold, and nickel were used for source and drain metallization.

Although many studies focus on common transistor parameters like charge carrier mobility, less deal with the Schottky contact and how it affects the performance of the TFTs. Therefore, we numerically fitted a mathematical model for Metal-Semiconductor-Metal (MSM) structures to the experimental data. In this way, we were able to extract the Schottky barrier height (SBH) and the ideality factors of the two Schottky junctions, which were formed from the contact between the source/drain and ZnO NP.

For all samples we have shown, the Schottky effect (or image force lowering effect) was presented, entailing a decreasing SBH with increasing drain-to-source voltage. An increasing positive gate voltage induced more charge carriers into the channel and caused the Schottky effect to be more prominent.

In general, nickel had the best performance among the three metallizations. For this metal and for higher drain-to-source voltages, the SBH tended to converge to some value around 0.3 eV, which could indicate possible fermi-level pinning.

## Figures and Tables

**Table 1:** TFT mobility (μ_e_ for electrons), subthreshold swing (SS), turn-on voltage (V_on_), and the ratio of the current in the on and off state (I_on_/I_off_) obtained from the transfer curves.

	V_on_ in V	I_on_/I_off_ Ratio	SS in V/dec	µ_e_ in cm^2^/Vs
Al L3	−0.60	2.2 × 10^3^	0.66	7.3 × 10^−4^
Al L5	−0.25	1.2 × 10^2^	1.39	2.1 × 10^−4^
Au L3	+1.1	3.0 × 10^3^	0.59	3.8 × 10^−2^
Au L5	−1.4	4.5 × 10^4^	0.55	1.3 × 10^−1^
Ni L3	−2.5	7.6 × 10^3^	0.60	2.0 × 10^−2^
Ni L5	−2.0	1.3 × 10^5^	1.08	1.5 × 10^−1^

## Data Availability

The data that support the findings of this study are available from the corresponding author upon reasonable request.
